# Supplementary material for: Phylogenetic Diversity of Wetland Plants across China
Source: Plants (Basel). 2021 Sep 6;10(9):1850. doi: 10.3390/plants10091850 (PMC8470339; doi:10.3390/plants10091850)
Supplement: Supplementary file 1 [file plants-10-01850-s001.zip › plants-1341499-supplementary/SM_20210729/SM_Figures.pdf]

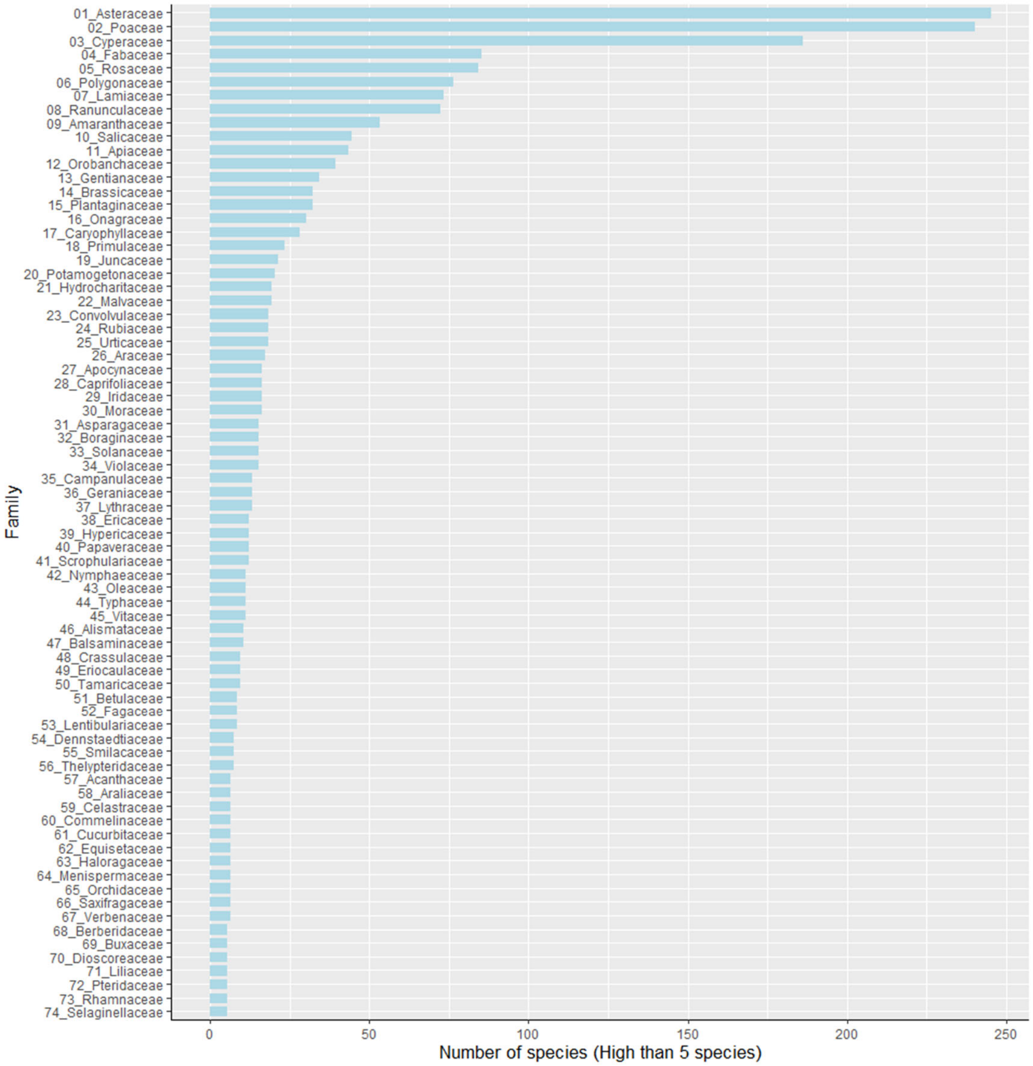

Figure S1 Number of species in each family, which had more than 5 species.

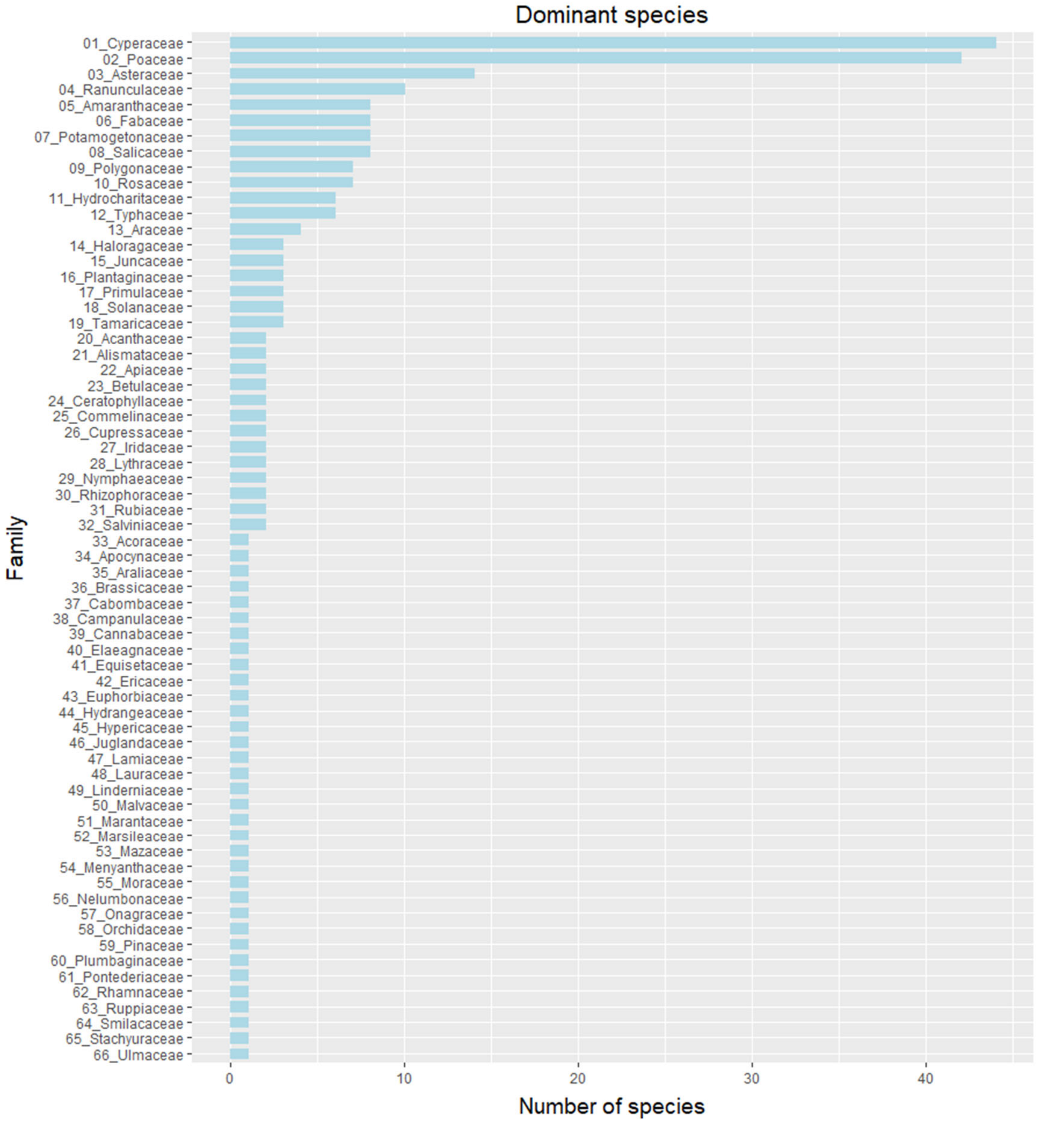

Figure S2 Number of species in each family of dominant species.

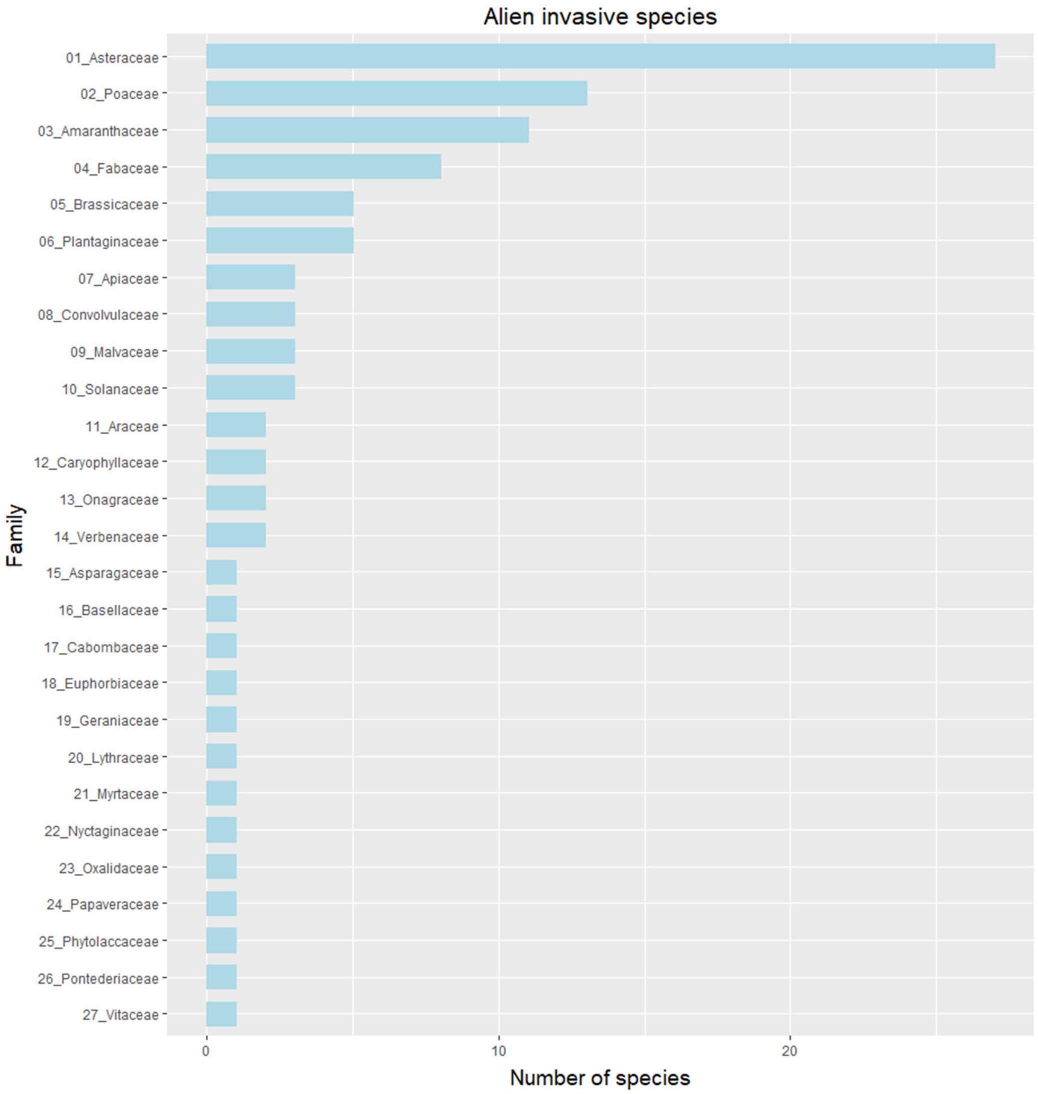

Figure S3 Number of species in each family of alien invasive species.

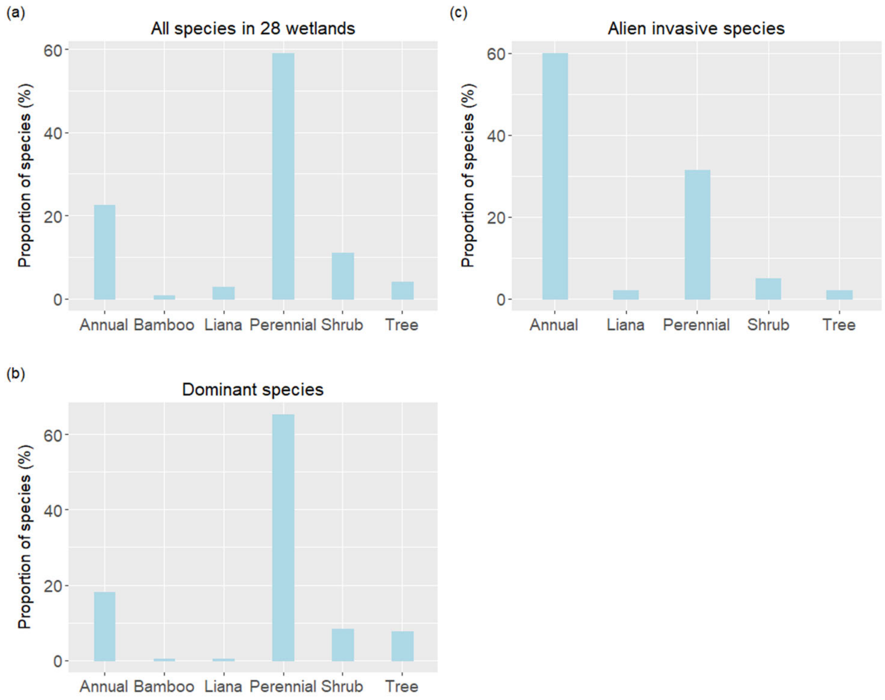

Figure S4 Proportions of wetland plant species of different life forms in 28 wetlands.

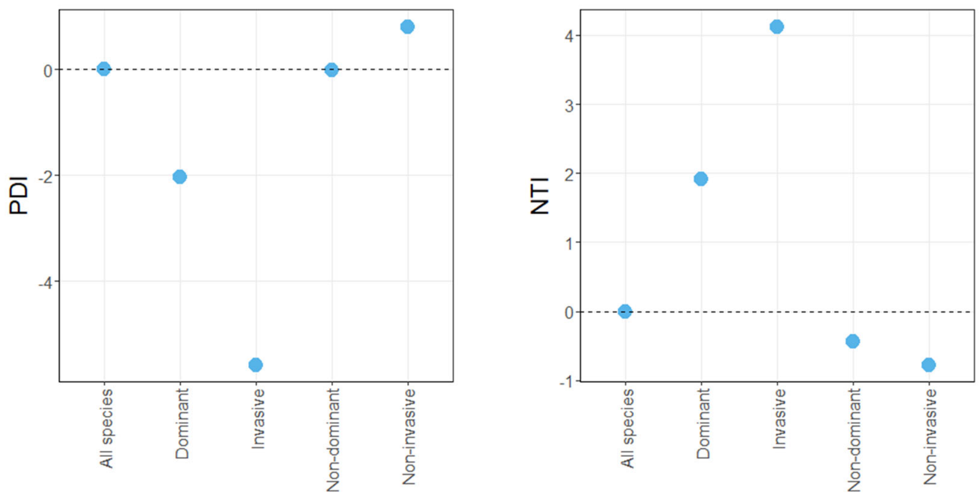

Figure S5 Phylogenetic diversity indices of different species assemblages in 28 wetlands.

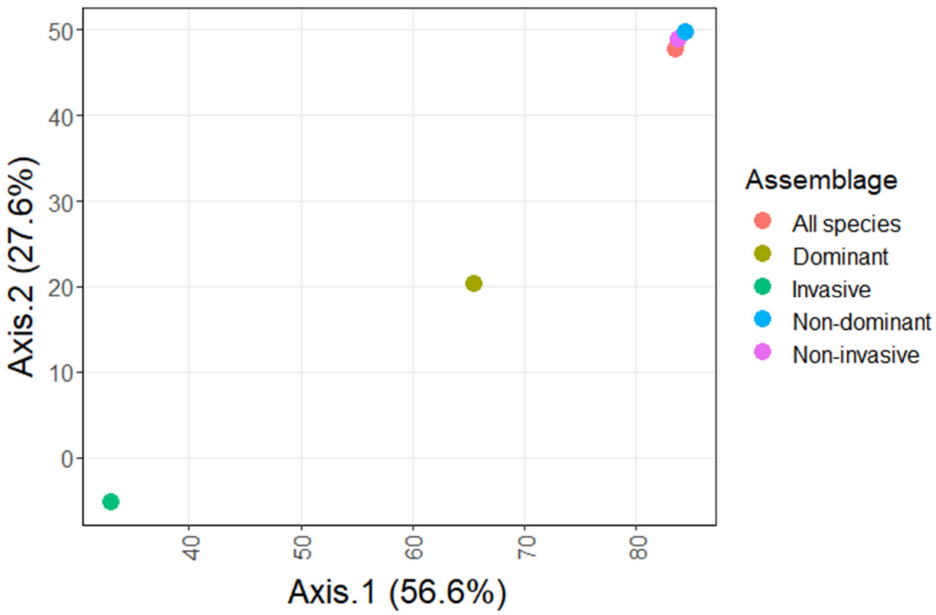

Figure S6 The mean phylogenetic distance to the nearest taxon for each taxon (MNTD) among species assemblages, visualized by a Principal Coordinates Analysis (PCoA) ordination biplot.
